# Structural specificity in plant–filamentous pathogen interactions

**DOI:** 10.1111/mpp.12983

**Published:** 2020-09-05

**Authors:** Aline Lacaze, David L. Joly

**Affiliations:** ^1^ Department of Biology Université de Moncton Moncton Canada

**Keywords:** developmental stages, filamentous pathogens, organ, plant disease, structural specificity, tissue

## Abstract

Plant diseases bear names such as leaf blights, root rots, sheath blights, tuber scabs, and stem cankers, indicating that symptoms occur preferentially on specific parts of host plants. Accordingly, many plant pathogens are specialized to infect and cause disease in specific tissues and organs. Conversely, others are able to infect a range of tissues, albeit often disease symptoms fluctuate in different organs infected by the same pathogen. The structural specificity of a pathogen defines the degree to which it is reliant on a given tissue, organ, or host developmental stage. It is influenced by both the microbe and the host but the processes shaping it are not well established. Here we review the current status on structural specificity of plant–filamentous pathogen interactions and highlight important research questions. Notably, this review addresses how constitutive defence and induced immunity as well as virulence processes vary across plant organs, tissues, and even cells. A better understanding of the mechanisms underlying structural specificity will aid targeted approaches for plant health, for instance by considering the variation in the nature and the amplitude of defence responses across distinct plant organs and tissues when performing selective breeding.

## Graphical abstract

Do plant resistance and pathogen virulence differ between organs, tissues, or even cell types? Many aspects of structural specificity are exposed in this review, as well as unknown elements that remain to be elucidated.

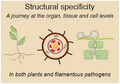

## 1 INTRODUCTION

**FIGURE 1:**
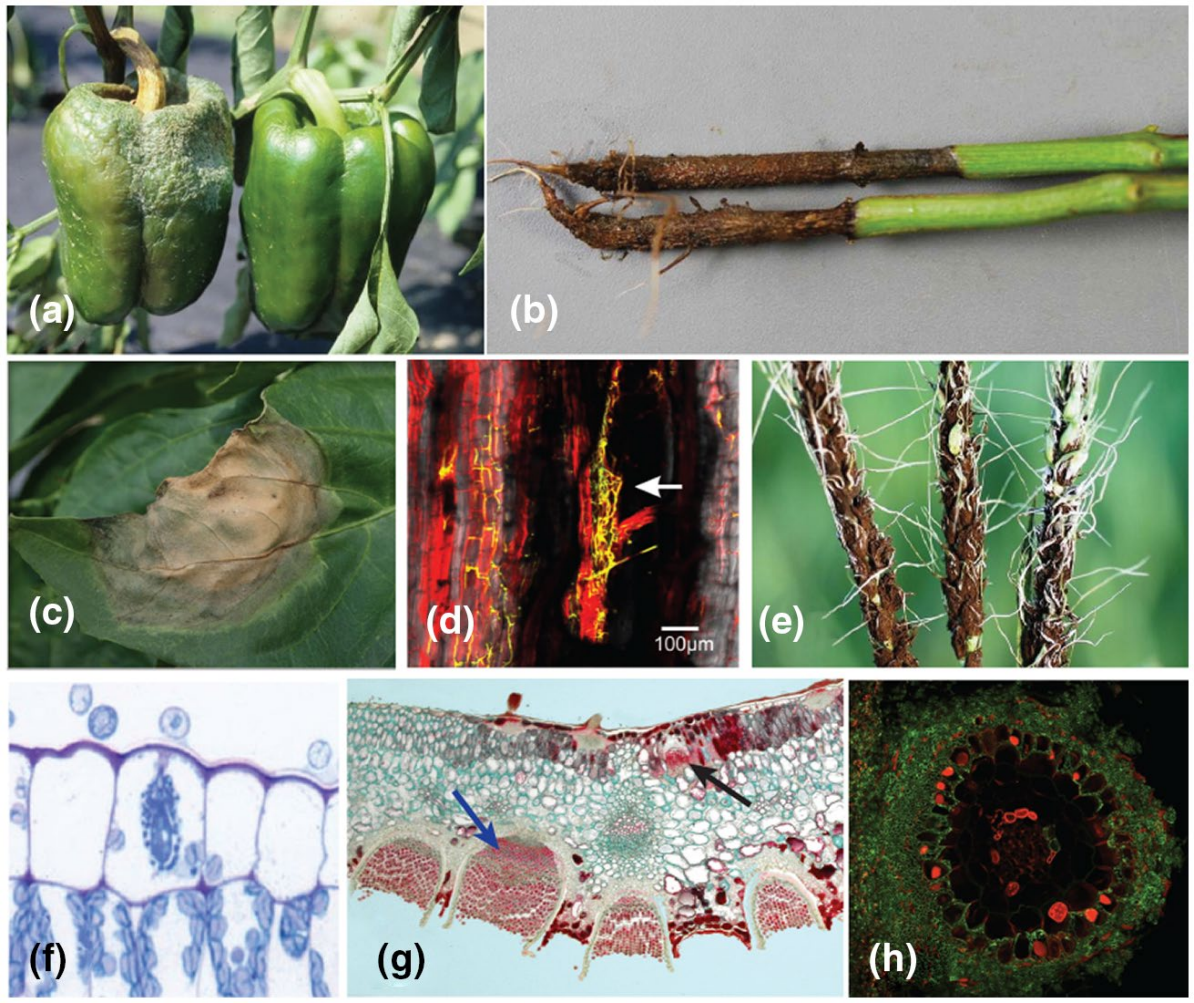
Examples of structural specificity among plant–microbe interactions. (a), (b), and (c) Considered a structural generalist, *Phytophthora capsici* can infect all organs of pepper (*Capsicum annuum*), including fruits (a), roots (b), and leaves (c), where resistance to each disease syndrome appears to be differentially inherited. Pathogens considered as structural generalists can sometimes infect all organs while being limited to certain tissues within these organs. Interestingly, this limitation sometimes changes in hemibiotrophic pathogens, depending on whether the pathogen is in its biotrophic or necrotrophic phase. For example, vascular pathogens such as *Fusarium oxysporum* will remain strictly limited to xylem tissues and surrounding cells as long as the plant is alive (d). Once the infected plant is killed, the fungus then invades parenchymatous tissues and sporulates on the plant surface. Other pathogens, often considered as extreme structural specialists, can infect most tissues asymptomatically, but will exhibit symptoms (or sporulate) only in highly specific tissues, such as anthers in the case of *Microbotryum* species and other anther smut fungi (e). Powdery mildews, such as *Golovinomyces cichoracearum*, are among the most extreme structural specialists, most species being confined to the epidermal cells of aerial plant parts (f). Rust fungi are also known as structural specialists, being only able to infect aerial organs. Interestingly, even though they can invade the whole mesophyll, many heteroecious rusts will produce pycnia specifically on the adaxial sides of leaves (black arrow) while aecia will be found on the abaxial side of leaves (blue arrow) (g). Structural specificity is not restricted to filamentous pathogens. Well‐known examples also exist among filamentous mutualists such as mycorrhizal fungi (e.g., *Laccaria bicolor*), which are restricted to root tissues up to the endodermis (h). Photograph credits: (a) Gerald J. Holmes, California Polytechnic State University; (b) Nancy Fisher Gregory, University of Delaware; (c) Christine Smart, Cornell University; (d) Guo *et al*. (2014) ; (e):Donald E. Groth, Louisiana State University, AgCenter; (f) Richard Bélanger and The American Phytopathological Society, (Wurms *et al*., 1999 ); (g) George L. Barron, University of Guelph Atrium Collection, https://atrium.lib.uoguelph.ca/xmlui/handle/10214/7059; (h) Clémence Bonnot and Francis Martin, INRA

Phytopathogens often establish and proliferate on specific plant organs and tissues, triggering leaf spots, stem cankers, fruit or root rots, and tuber scabs. A pathogen's structural specificity is often caused by adaptation to a given organ (leaf, stem, flower, root, etc.), tissue (xylem, phloem, mesophyll, etc.), or a developmental stage of the host (Barrett and Heil, 2012). Such specificity can be expressed at the level of its entry tissue, its colonized tissue, its sporulating tissue, or any combination of these factors. Pathogens can exert high structural specificity, such as powdery mildew species confined to the leaf epidermis, or have no structural specificity, such as *Sclerotinia sclerotiorum*, a generalist capable of infecting virtually any tissue. Furthermore, a pathogen's structural specificity can change throughout its lifecycle (e.g., Figure 1g,h). Both structural specialists and generalists may have benefits considering infection strategies. Examples of such advantages include performance, competition, or niche expansion (Barrett and Heil, 2012).

However, research on the processes driving structural specificity is still in its infancy and many questions are only partially answered. How can plant pathogens adapt to various plant structures? How do plant–microbe interaction‐relevant processes differ between plant structures? Recent reports indicate that various plant pathogens, whether bacteria, fungi, or oomycetes, require adaptations to efficiently infect and colonize given tissues of their hosts (Barrett and Heil, 2012; Fatima and Senthil‐Kumar, 2015; Strugala *et al*., 2015; Chuberre *et al*., 2018). In parallel, evidence of structure‐specific plant resistance is accumulating, and involves both constitutive and induced defences. Here, we summarize relevant knowledge on the structural specificity of plant–filamentous pathogen interactions. While such interactions are the results of complex coevolutionary patterns, aspects influencing a pathogen's structural specificity will be separately presented, first in the context of plant defences and then with regards to pathogen virulence.

## 2 STRUCTURAL SPECIFICITY OF PLANT RESISTANCE AND SUSCEPTIBILITY

Quantitative differences of susceptibility/resistance outcomes in different organs, tissues, and cells can drive the degree of specialization of a given pathogen. Organ‐specific architectures such as air spaces and stomata, tissue properties such as a waxy cuticle or a lignified endodermis, as well as cellular responses mediated by cell surface or intracellular immune receptors altogether contribute, and a combination of different factors might lead to full resistance towards a pathogen that has not evolved specific mechanisms to infect those structures. For example, the success of leaf infection by powdery mildew species is not informative of the resistance status of the roots of the same plant, as the unsuccessful outcome in this different organ could be due to the pathogen being nonadapted to infect this organ. Thus, organisms considered as generalist, but exhibiting various degrees of organ or tissue specificity are probably more suited to the study of structural specificity as they allow the comparison of successful interactions.

Importantly, the resistance status of an organ does not necessarily determine the level of compatibility of a whole plant to a pathogen. This has been highlighted in potato, whose resistance in leaves does not always correlate with tuber resistance to *Phytophthora infestans*. As early as 1999, Oberhagemann *et al*. detected a quantitative trait locus (QTL) on chromosome V that was associated with foliar resistance, but also with susceptibility in tubers. Quantitative resistance loci (QRLs) associated with resistance in the foliage have also been shown to be largely distinct from QRLs associated with resistance in tubers (Mayton *et al*., 2010). Similarly, no correlation is observed between disease scores in roots and leaves of barley accessions infected with *Phytophthora palmivora*, even though it exhibits its typical hemibiotrophic lifestyle during infection of both organs (Le Fevre *et al*., 2016). Thus, the outcome of the interaction between a plant and a specific pathogen, that is, the degree of susceptibility, can clearly differ depending on the structure being infected.

At the cell level, three layers of plant defence are distinguished in plant–pathogen interactions: preformed defences, induced immunity mediated by cell membrane‐associated pattern recognition receptors (PRRs), and induced immunity mediated by intracellular nucleotide‐binding domain leucine‐rich repeat proteins (NLRs). In this review, we opted for a receptor‐based nomenclature instead of the classical PAMP‐triggered and effector‐triggered immunity (PTI and ETI) dichotomy that is sometimes considered confusing and/or controversial (Thomma *et al*., 2011; Kanyuka and Rudd, 2019). In the first part of this review, we will focus on differences observed between plant structures (i.e., preformed mechanisms or its components with tissue‐ or organ‐specific distribution) in the context of plant–microbe interactions.

### 2.1 Preformed defences

Preformed defences are constitutive and encompass physical and chemical barriers to phytopathogens. As aboveground and belowground environments differ, it is conceivable that preformed barriers differ as well. For instance, the formation of a cuticle is limited to aerial plant organs and the emerging root tips (Berhin *et al*., 2019). The cuticle may play a role in the organ specificity of the pea–*Nectria haematococca* interaction, as demonstrated by Schäfer and Yoder (1994). Indeed, they depicted that *N. haematococca* can attack roots and the stem base but not the leaves of pea, unless those leaves are wounded, in which case it rapidly colonizes the aerial parts of pea. Developed plant roots lack a cuticle layer, which might explain why *N. haematococca* can directly infect roots but not leaves. Similarly, while *P. palmivora* can infect leaves and roots of barley, it cannot penetrate the leaf epidermis, but only enters through wounds (Le Fevre *et al*., 2016). Nevertheless, most plants harbour roots that also possess distinct specific physical protective barriers, the exodermis and the endodermis. Both of these internal cell layers are usually surrounded by Casparian strips, root‐specific boundaries mainly composed of lignin (De Coninck *et al*., 2015). In brief, the cuticle found in aerial plant parts and the lignified layers found in underground organs are both physical barriers that limit pathogen invasions (Nawrath *et al*., 2013).

#### 2.1.1 Organ‐specific constitutively expressed pathogenesis‐related proteins

De Coninck *et al*. (2015) described evidence that pathogenesis‐related (PR) proteins exhibit organ specificity. Even in healthy plants, many PR proteins accumulate specifically in certain organs or developmental stages (van Loon *et al*., 2006). For instance, β‐glucanases (PR‐2) and chitinases (PR‐3) are not detectable in young tobacco leaves of noninfected plants but do accumulate constitutively in roots (Broekaert *et al*., 2000). Accordingly, a constitutive abundance of PR‐2 and PR‐3 has been observed in root exudates of *Arabidopsis thaliana*, which appears to be developmentally regulated (De‐la‐Pena *et al*., 2010). Thus, certain PR proteins appear to be constitutively produced in an organ‐specific manner to ensure disease prevention by the plant. PR protein expression in specific organs may reflect adaptations to different environments harbouring different microbial communities. In the case of pathogenic fungi on leaves, the absence of β‐glucanases and chitinases in leaves may be due to the preformed cuticle barrier, which does not necessitate further costly expression of additional defences.

#### 2.1.2 Differential distribution of secondary metabolites

It has been suggested that roots and leaves differ greatly with regard to all major soluble phenylpropanoid constituents. Indeed, *A. thaliana* roots contain three prominent phenylpropanoids (coniferin, syringin, and scolopin) that are not found in leaves (Bednarek, 2005). Those phenylpropanoids have been classified as putative preformed defence‐related compounds, and have been postulated to act as storage and/or transport forms of lignin precursors (Boerjan *et al*., 2003). Such contrasted abundances of secondary metabolites might reflect differences in production costs between organs or relate to a need to isolate those compounds from other constituents.

Similarly, two antimicrobial compound families, glucosinolates and saponins, are subject to organ and tissue specificity (Osbourn, 1996). Indeed, in oilseed rape, aliphatic glucosinolates predominate in leaves, while indolyl and phenylethyl glucosinolates are the major glucosinolates in roots and stems. Similarly, the distribution of two types of saponins is mutually exclusive: avenacins are predominantly found in roots while avenacosides are mostly located in shoots and leaves. Even within these organs, these compounds are more abundant in the outer cell layers, with avenacin being restricted to root epidermal cells and avenacosides being present at higher concentrations in the epidermis compared to the rest of the leaf. These secondary metabolites probably act as the first line of defence by accumulating in cell layersthat are most prone to attack.

Such organ and tissue specificity of antimicrobial compounds is also observed in solanaceaous species. Indeed, the antimicrobial sesquiterpenoid phytoalexin capsidiol is thought to be organ specific in *Nicotiana*, where it is constitutively expressed in roots but not detectable in shoots (Bohlmann *et al*., 2002). However, capsidiol is induced in both organs after wounding or infection by pathogens. Thus, the structural specificity of defence factors should not be seen as static, but rather as an adaptive mechanism that may fluctuate following developmental signals or exposure to stresses.

Similarly, antimicrobial compounds such as tropane alkaloids and nicotine often exhibit organ‐ or tissue‐specific synthesis, after which they can be transported to other plant organs. For instance, nicotine is only biosynthesized in *Nicotiana tabacum* roots besides a cell specificity of metabolic compounds production (Colinas and Goossens, 2018). Another example concerns the tuber peel of *Solanum tuberosum*, which exhibits higher concentrations of calystegine, a tropane alkaloid, compared to other tuber tissues (Keiner and Dräger, 2000).

### 2.2 PRR‐mediated immunity

Another component of plant defence is PRR‐mediated immunity (PMI), often induced following pathogen‐associated molecular patterns (PAMPs) recognition, but not solely (e.g., *Cladosporium fulvum* avirulence proteins also trigger PMI). This perception involves the activation of PRRs, a group of proteins encompassing receptor‐like proteins (RLPs) and receptor‐like kinases (RLKs). Recognition by PRRs usually induces rapid defence responses such as the production of jasmonic acid (JA) and/or salicylic acid (SA), the production of reactive oxygen species (ROS), and/or the expression of PMI‐associated genes such as PR proteins, lipoxygenases, and WRKY transcription factors. In *A. thaliana*, many RLKs are under the control of tissue and developmental stage‐specific promoters and thus display distinct spatial patterns across plant organs (Wu *et al*., 2016). As RLKs also play roles in various plant physiological processes, their tissue specificity might reflect their localized roles in functions not directly related to immunity. However, it has been demonstrated that the receptor of the bacterial flagellin epitope flg22 (FLS2) is active in a cell type‐ and tissue‐specific manner in *A. thaliana* (Millet *et al*., 2010; Beck *et al*., 2014). Other examples of structure‐specific RLKs involved in plant immunity can be found in *A. thaliana*, such as PEPR1, PEPR2, and SRF1 (Eyüboglu *et al*., 2007; Bartels *et al*., 2013), in *Brassica napus*, for example SERK1 and SERK2 (Ahmadi *et al*., 2016), or in *Malus domestica*, for example WAK‐RLK (Zuo *et al*., 2019; see Table 1).

**TABLE 1:** Selected plant and pathogen factors contributing to structural specificity and discussed in this review

	Plant factors	Plant host	Predominant localization	References
**Pre‐infection**				
Quantitative resistance	QTLs (late blight resistance)	*Solanum tuberosum* (chr. V)	Leaves	Oberhagemann *et al*. (1999)
	QRLs (late blight resistance)	*S. tuberosum*	Leaves and tubers (distinct)	Mayton *et al*. (2010)
Physical barriers	Cuticle	Any land plant	Leaves and root tips	Berhin *et al*. (2019)
	Casparian strip	Any land plant	Roots	De Coninck *et al*. (2015)
Constitutive PR proteins	*PR‐2* (β‐glucanases)	*Nicotiana tabacum*	Roots	Broekaert *et al*. (2000)
	*PR‐3* (Chitinases)	*N. tabacum*	Roots	Broekaert *et al*. (2000)
Constitutive secondary metabolites	Phenylpropanoids^a^	*Arabidopsis thaliana*	Roots	Bednarek (2005)
	Glucosinolates^a^	*Brassica napus*	Roots and stems	Osbourn (1996)
	Saponins^a^	*B. napus*	Roots or shoots	Osbourn (1996)
**Early infection**				
RLKs	*SERK1*	*B. napus*	Developped shoots	Ahmadi *et al*. (2016)
	*SERK2*	*B. napus*	Shoots	Ahmadi *et al*. (2016)
	*PEPR1*	*A. thaliana*	Vascular tissues	Bartels *et al*. (2013)
	*PEPR2*	*A. thaliana*	Vascular tissues	Bartels *et al*. (2013)
	*SRF1*	*A. thaliana*	Vascular tissues	Eyüboglu *et al*. (2007)
	*MDP0000232725* (WAK‐RLK)	*Malus domestica*	Roots	Zuo *et al*. (2019)
				
	**Pathogen factors**	**Pathogen**	**Predominant localization**	**References**
Nutrient acquisition	*NRT* (nitrate transporter)	*Phytophthora infestans*	Potato leaves	Abrahamian *et al*. (2016)
Morphological adaptation	*BUF1* (melanin synthesis)	*Magnaporthe oryzae*	Mutant penetrates roots but not leaves	Sesma and Osbourn (2004)
	*FOW1* (mitochondrial carrier)	*Fusarium oxysporum*	Mutant infects roots but not vasculature	Inoue *et al*. (2002)
**Late infection**				
R‐genes	*Rpi‐blb1 (RB)*	*S. tuberosum*	Leaves	Gao and Bradeen (2016)
	*R2*	*S. tuberosum*	Leaves	Roer and Toxopeus (1961)
	*R3a*	*S. tuberosum*	Leaves	Park *et al*. (2005)
	*Rpi‐abpt*	*S. tuberosum*	Leaves	Park *et al*. (2005)
	**Pathogen factors**	**Pathogen**	**Predominant localization**	**References**
Effectors	*um02239* (and 6 others)	*Ustilago maydis*	Leaves	Schilling *et al*. (2014)
	*um05439* and *um03650*	*U. maydis*	Tassels	Schilling *et al*. (2014)

^a^ Specific metabolite among those categories was found to be organ‐specific.

#### 2.2.1 PMI‐responses induced by PAMPs

Plant defences triggered by PMI can be investigated via the use of synthetic PAMPs such as the flg22 and elf18 peptides (the active epitopes of bacterial flagellin and EF‐Tu [elongation factor Thermo Unstable], respectively). Even though flg22 and elf18 are bacterial PAMPs not present among filamentous pathogens, they are the most studied PAMPs, their mechanisms of action and recognition have been well characterized, and it is largely assumed that perception of fungal and oomycete PAMPs would behave similarly. Recent investigations suggested perception of flg22 and elf18 may have a different impact on the shoot and root growth. Ranf *et al*. (2011) performed growth inhibition assays of *A. thaliana* Col‐0 seedlings on flg22‐ or elf18‐containing agar plates. They observed that seedlings on flg22‐containing plates had shorter roots with many lateral roots, while the shoots were smaller, sometimes with slightly yellowish leaves. By contrast, seedlings on elf18‐containing plates had tiny, dark brown cotyledons but long, thin primary roots without lateral roots.

Wyrsch *et al*. (2015) investigated *A. thaliana* responses following elicitation by flg22 or elf18 in roots and leaves. They observed that isolated wild‐type root tissue is able to induce PMI responses such as ROS production and MAPK activation upon flg22 perception, in contrast to elf18. All root tissues appeared able to perceive flg22 and induce PMI responses when FLS2 was present. However, the intensity of the immune responses did not always correlate with the expression level of FLS2 but rather depended on the expressing tissue, demonstrating that the induction of defence responses to flg22 in roots fluctuates spatially. In filamentous pathogens, chitin is an essential component of the fungal cell wall (Lenardon *et al*., 2010) that has been shown to elicit tissue‐specific PMI. Indeed, chitin induces callose deposition in the mature zones of *A. thaliana* roots, but not in the elongation zones (Millet *et al*., 2010). This supports the idea that differential PAMP perception and sensitivity might contribute to a proper balance of defence responses between tissues according to their expected exposure to elicitors. These findings support the hypothesis that, in natural conditions, plants might need to restrict the expression of PAMP receptors to tissue‐specific locations, especially at putative pathogen entry sites, in order to efficiently inhibit pathogen invasion and regulate PMI signalling.

#### 2.2.2 Hormonal signalling pathways in PMI

In some cases, extrapolation of findings from one plant organ to another might lead to misleading generalizations. This holds true for SA and JA signalling pathways. It has been reported that SA, which is considered a requirement for basal defence in leaves against biotrophic pathogens, does not appear to be as important for root immune responses (Jones and Dangl, 2006; Millet *et al*., 2010; Munch *et al*., 2018). During *A. thaliana* leaf infections by *Phytophthora parasitica*, a hemibiotrophic oomycete, transition from biotrophy to necrotrophy is usually correlated with a shift of plant defences from SA‐ to JA‐mediated responses (Glazebrook, 2005; Attard *et al*., 2010). However, marker genes of both SA‐ and JA‐dependent pathways are transiently expressed during penetration of root tissues by *P. parasitica* (Attard *et al*., 2010), suggesting that JA and SA signalling pathways act in synergy in *A. thaliana* roots infected by *P. parasitica*. Similar cooperative effects of JA and SA have been observed during *A. thaliana* roots infection by the fungus *Fusarium oxysporum* (Berrocal‐Lobo and Molina, 2008). These findings contrast with the reported antagonistic action of the signalling pathways involving SA and JA in leaves (Glazebrook, 2005). Thus, the relevance of findings obtained by studying a particular organ might be limited to that organ, showing the need for additional studies before blindly extending knowledge acquired in one organ to other organs or tissues.

#### 2.2.3 Pathogenesis‐related proteins and induced secondary metabolites

In addition to their differential constitutive accumulation in specific tissues of healthy plants (discussed above), induction of PR protein expression upon infection can also occur in an organ‐specific manner. *F. oxysporum* infects roots before spreading through the root vasculature to cause disease in shoots. Chen *et al*. profiled the expression of *A. thaliana* root genes after infection with *F. oxysporum* and contrasted it to leaf infection (Chen *et al*., 2015). In contrast to leaves, there was a relative absence of defensin and PR protein gene expression in infected root tissues.

In the *F. oxysporum*–cotton interaction, there is little overlap in the identity of the differentially expressed genes detected in infected root and hypocotyl tissues, indicating that overall the tissues respond quite differently to infection (Dowd *et al*., 2004). In contrast with leaves, defence‐related genes are constitutively expressed in roots and do not show significant induction following infection. Furthermore, homologues of genes associated with tannin, anthocyanin, and lignin biosynthesis are repressed in infected root tissues of cotton. As lignin production is a defence response to pathogen, they suggest that repression of this biosynthesis may be representative of the pathogen counteract of defence responses in cotton roots. In contrast, in the hypocotyl tissues, genes associated with phenylpropanoid, flavonoid, and terpenoid indole alkaloid biosynthesis are induced in response to infection.

### 2.3 NLR‐mediated immunity

NLR‐mediated immunity (NMI) represents a strategy to respond to intracellular microbial signals (as opposed to extracellular microbial signals in PMI), usually induced by the recognition of effectors or their effect on host targets. Effectors are proteins secreted by pathogens that alter plant processes to favour infection. They can be detected by cytoplasmic proteins encoded by NLR genes (nucleotide‐binding leucine‐rich repeat), leading to NMI, which often results in the hypersensitive response (HR), a form of highly localized programmed cell death.

#### 2.3.1 Differential inheritance of resistance: organ‐specific genetic mechanisms?

An example of the organ‐specific nature of resistance has been reported through the investigation of the *Phytophthora capsici*–pepper pathosystem (Walker and Bosland, 1999). It was observed that resistance to root rot and foliar blight depended on different genetic mechanisms. Further comparative studies not only confirmed these results but demonstrated that the inheritance of resistance to stem blight was also distinct, with ratios consistent with single genes controlling resistance to each disease (Sy *et al*., 2005). Thus, breeding for resistance against *P. capsici* implies dealing with at least three sets of genetic determinants in the same plant. In *P. infestans* and *Phytophthora sojae* all effectors demonstrated to trigger immunity belong to the RXLR class (Yin *et al*., 2017; Zhang *et al*., 2019, Lin *et al*., 2020), and their plant counterparts, that is, the proteins that recognize them, all belong to the NLR class (Zhang *et al*., 2013; Vossen *et al*., 2016). It is thus likely that additional gene‐for‐gene interactions between other *Phytophthora* species and their host plants are dictated by the same types of proteins. Indeed, *P. capsici* also appears to carry a large repertoire of RXLR effectors (Lamour *et al*., 2012), and one of them (PcAvh1) has recently been characterized in the context of its role in virulence (Chen *et al*., 2019). Resistance to fruit rot of pepper is completely different compared to other *P. capsici* syndromes on pepper as it is not resistance (R)‐gene based but rather correlated with organ maturity (Foster and Hausbeck, 2010; Naegele *et al*., 2014). Similarly, resistance of cucurbit fruits to infection by *P. capsici* also appears to be related to physiological changes associated with fruit development and thus increases with maturity (Ando *et al*., 2009). While the susceptibility status of an organ might be affected by its developmental stage, there does not seem to be a clear direction (both age‐related resistance and mature tissue susceptibility have been evoked in different studies; Le Fevre *et al*., 2016).

**FIGURE 2:**
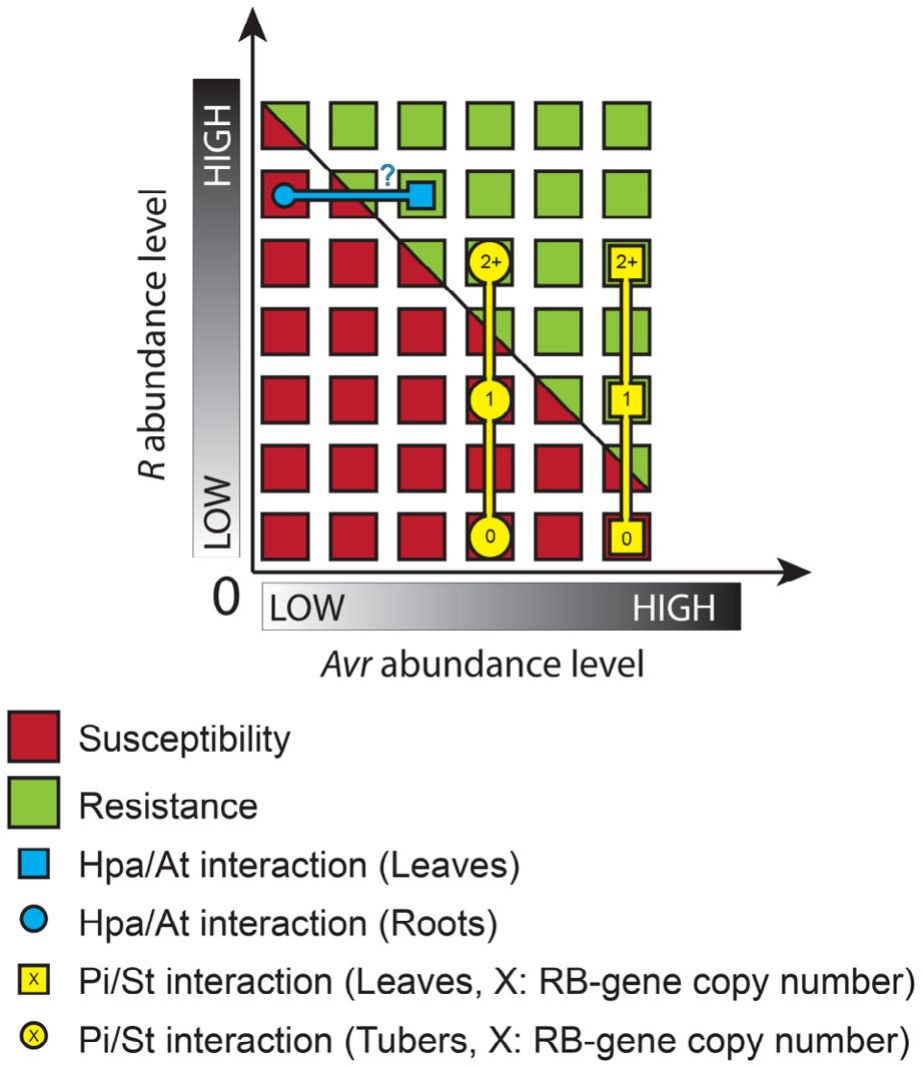
Resistance and susceptibility in a plant–pathogen interaction depend on the abundance levels of both R and Avr factors, which can vary between organs. Blue elements: *Hyaloperonospora parasitica–Arabidopsis thaliana* interaction. For a given R‐gene expression level, *A. thaliana* roots are susceptible whereas leaves are resistant to *H. parasitica* (Hermanns *et al*., 2003). This contrasted phenotype may rely on distinct Avr gene expression in leaves and roots from the pathogen. Yellow elements: *Phytophthora infestans*–*Solanum tuberosum* interaction. In this context, a given level of expression of the R*‐*gene (e.g., from one copy of the gene *RB*) might be sufficient to trigger a hypersensitive reaction in leaves, where the cognate Avr is highly expressed. However, it might prove insufficient in tubers where the Avr could be weakly expressed, a situation where a higher level of expression of *RB* (e.g., from multiple copies of it as a transgene) could thus complement this difference to achieve the same outcome (resistance; Gao and Bradeen, 2016). Note: Abundance can fluctuate for various reasons, whether expression levels or the existence of distinct delivery mechanisms and/or infection structures

*Hyaloperonospora arabidopsidis* is the oomycete causing downy mildew in *A. thaliana*, a disease typically recognized to affect leaves. In this pathosystem, incompatible host–pathogen combinations show the expected outcomes (in terms of oxidative burst and HR) in leaves, while roots show no signs of active defence and appear completely susceptible to any *H. arabidopsidis* isolates (Hermanns *et al*., 2003). Quantitative reverse transcription PCR (RT‐qPCR) showed that the R‐gene *RPP1*, which mediates resistance in leaves to *H. arabidopsidis* isolates harbouring the avirulence allele of *ATR1*, is expressed in leaves as well as in roots. Similarly, *NDR1* and *EDS1*, two components of NLR‐mediated signalling pathways, were also expressed in both tissues, demonstrating that expression of R‐genes and downstream components of the signalling cascade is not sufficient for the induction of avirulence gene‐mediated defence mechanisms in roots. An explanation suggested by the authors for the lack of induction of R‐gene dependent responses in roots was that other essential downstream signalling components might be expressed in an organ‐ or tissue‐specific manner and thus absent in roots. They hypothesized that a so far unidentified “enabler of defence responses” could be lacking in roots but present in leaves. Another hypothesis, not suggested by the authors, would be that the absence of resistance comes from the absence of expression (or reduced expression) of the cognate avirulence gene *ATR1* (see Figure 2).

In the potato late blight pathosystem, dominant R‐gene resistance is often thought to be effective in both foliage and tubers, as is the case for *R1* and *Rpi‐phu1* (Park *et al*., 2005; Śliwka *et al*., 2006). However, there are other cases in which the R‐gene is not effective in tubers, such as *Rpi‐blb1* (Halterman *et al*., 2008), *R2*, *R3a*, and *Rpi‐abpt* (Roer and Toxopeus, 1961; Park *et al*., 2005). Interestingly, preliminary results (Sormany, 2018) indeed suggest that the efficiency of R‐genes in a given organ is somehow correlated with the expression levels of the corresponding avirulence genes in that organ, a hypothesis that could explain the results of multiple studies (Figure 2). In *P. infestans*, expression of Avr genes is closely linked to the formation of haustoria. Thus, it remains to be determined whether the Avr expression is truly different in leaves compared to tubers or whether this could be linked with a lower abundance of haustoria in tubers.

#### 2.3.2 Organ specificity of NLR signalling

Several NLR genes are known to display organ‐specific expression patterns, as has been demonstrated following a meta‐analysis focusing on expression data of 1,235 NLRs from nine plant species (Munch *et al*., 2018). As stated by the authors, NLR expression probably reflects organ‐specific differences in effector recognition. Interestingly, they found root:shoot expression ratios to be stable within species, suggesting organ‐specific hardwiring of NLR expression patterns matching distinct challenges. Indeed, most monocot and dicot plant species preferentially expressed NLRs in roots. However, brassicaceaous species, including oilseed rape (*Brassica napus*) and *A. thaliana*, were unique in showing NLR expression skewed toward shoots across multiple phylogenetically distinct groups of NLRs. The overall root‐skew in NLR expression of the majority of plant species suggests that roots probably experience a higher level of effector pressure than shoots, despite NLR function having mainly been characterized in the context of shoot–pathogen interactions. This NLR expression shift suggests that the members of the Brassicaceae family may have evolved unique PRRs and antimicrobial root metabolites to substitute for NLR protection and an unusual mode of plant–microbe interaction for this plant family (also reflected in the inability of certain Brassicaceae members to establish relationships with mycorrhizal fungi (Delaux *et al*., 2014).

#### 2.3.3 Variation in gene dosage and expression levels

Another study compared the differences and similarities of defence mechanisms against *P. infestans* in potato foliage and tubers (Gao and Bradeen, 2016). By investigating potato transgenic lines, they demonstrated that higher gene copy numbers of *RB* (i.e., the R‐gene also known as *Rpi‐blb1*), and thus higher expression levels of *RB*, are correlated with increased resistance in both foliage and tubers. Interestingly, the gene dosage requirements to attain resistance appeared different between leaves and tubers. While transgenic plants harbouring a single copy of the gene are resistant at the foliar level, multiple copies of the gene are needed to confer resistance in tubers. The authors concluded that R‐gene dosage and the resulting variation in R‐gene transcript levels may be determining factors in whether disease resistance is manifested in an organ‐specific manner. Again, another possibility would be that a combination of dosage and/or expression of both R‐ and Avr‐genes dictates the outcome of an interaction (Crute and Norwood, 1986), and that the dosage of each member could dictate organ‐specific patterns (Figure 2).

## 3 STRUCTURAL SPECIFICITY OF PATHOGEN INFECTION

Some pathogens are not restricted to a given plant organ and are able to infect any plant tissues. Considering the distinct architecture of various plant tissues and the fact that nutrient distribution is not equal among them, it is highly conceivable that pathogens adapt their physiological processes and metabolism when infecting distinct organs. In some cases, such adaptations might lead the pathogen to deploy organ‐specific structures. In parallel, virulence factors (effectors) can be deployed by plant pathogens in an organ‐specific manner as they acquire their nutrients to sustain parasitic colonization and complete their life cycle. Here we discuss the extent of structure‐specific factors in pathogen virulence and in which form they appear.

### 3.1 Structural‐specificity and nutrient availability/acquisition

Sugars and amino acids are mainly present in the phloem and the leaf apoplast whereas mineral nutrients and water are abundant in the xylem and the root apoplast (Dinant *et al*., 2010; Myburg *et al*., 2013). Some studies suggest that bacterial pathogens have evolved nutrient acquisition strategies based on the nutrient abundance in the host plant, together with chemotaxis and motility that could play a role in recognition of nutrient niches and their colonization (Fatima and Senthil‐Kumar, 2015). Differences in nutrient use among pathogen developmental stages thus probably reflect the encountered tissues (e.g., penetration of the epidermal layer versus colonization of mesophyll or cortical tissues). Indeed, lipids are the primary sources of energy during germination and penetration (Solomon *et al*., 2003). They are required to generate the osmotic pressure for penetration. Carbohydrates and proteins are used sparingly at this early stage. After penetration, sugars are available and become the main energy source, whilst lipolysis is inhibited. Plant pathogen adaptation to available nutrients has been highlighted in the potato late blight pathosystem (Abrahamian *et al*., 2016). Indeed, the versatility of *P. infestans* as a pathogen of both leaves and tubers is reflected in the organ‐specific expression patterns of its transporters. For example, the mRNA level of a nitrate transporter (NRT) is about 100‐fold higher during mid‐infection in leaves (a nitrate‐rich organ) compared to tubers (a nitrate‐poor organ). Interestingly, mutants with a reduced ability to assimilate nitrate were nonpathogenic on leaves but successfully colonized tubers, suggesting a link between nutrient acquisition and virulence.

The sensing of carbon sources has been suggested to guide developmental shifts in *Ustilago maydis* during pathogenesis. By sensing plant surface cues such as hydrophobicity or the presence of cutin monomers, this fungus alters the expression of several genes to initiate its biotrophic infection. Subsequently, transitions in carbon acquisition occur between penetration and growth in the leaf mesophyll relative to the moment *U. maydis* reaches the vasculature and stimulates cell morphology changes (Lanver *et al*., 2014). Interestingly, carbon metabolism transitions are linked to shifts in effector gene expression. Goulet and Saville (2017) hypothesized that a common control mechanism might exist, or that carbon sensing might direct a shift in carbon metabolism and in effector expression to facilitate access to new carbon sources. More recently, Lanver *et al*. (2018) suggested that the expression of several transporters appears to be linked with “virulence modules” (i.e., developmental stages) rather than being controlled directly by nutrient availability. Another study suggested that aggressiveness of plant pathogens is shaped by the constitutive plant defence level rather than nutrient availability (Maupetit *et al*., 2018). Thus, nutrient availability and acquisition might partially determine the structure specificity of pathogen virulence in some pathosystems, but other factors, such as pathogen developmental stages, are clearly of importance in other pathosystems.

### 3.2 Colonization of distinct tissues can be associated with distinct developmental stages of pathogens

The coffee leaf rust fungus (*Hemileia vastatrix*) is known to produce pioneer haustoria in coffee stomatal subsidiary and guard cells, followed by secondary haustoria in mesophyll cells. Interestingly, recognition of the fungus never occurs at the pioneer haustoria stage, suggesting that avirulence factors are not recognized by, or secreted into, stomatal cells (Ramiro *et al*., 2009). Similarly, the fungus *Leptosphaeria maculans* infects *B. napus* in two stages: infection of cotyledon, followed by colonization of the stem. These stages have been associated with different waves of effectors in *L. maculans* (Gervais *et al*., 2017). Thus, developmental stages of plant pathogens can be intimately linked to structural specificity through the deployment of effectors. Effectors secreted by appressoria and intracellular hyphae can also be spatially segregated in terms of infected tissues. Indeed, in the *Colletotrichum higginsianum*–*A. thaliana* pathosystem effectors secreted by appressoria are localized in epidermal cells while those secreted by intracellular hyphae are localized in mesophyll cells (Kleemann *et al*., 2012). In such cases, so‐called waves of effectors can be considered tissue specific.

### 3.3 Morphological adaptation to plant organs

As reported earlier, *P. capsici* causes several disease syndromes in pepper (Sy *et al*., 2005), where it differentially infects the distinct plant organs. Irwin *et al*. (1997) reported that pepper plants affected by root rot lack conspicuous appressoria, whereas in the case of foliar blight the pathogen produces pronounced appressoria. Conversely, *P. palmivora* forms appressoria when colonizing barley cv. Baronesse roots, but not when colonizing leaves, unless they have been pinpricked (Le Fevre *et al*., 2016). When infecting liverworts, *P. palmivora* invades air chambers of the dorsal photosynthetic layer (a tissue not present in vascular plants), where it develops tissue‐specific haustoria‐like infection structures (Carella *et al*., 2018).

In several plant pathogenic fungi, formation of tissue‐ or organ‐specific infection structures is also observed. For instance, when *Colletotrichum graminicola* colonizes maize roots, it forms structures that are commonly found in root pathogens, including hyphopodia, runner hyphae, and microsclerotia, that are not found during foliar infections (Sukno *et al*., 2008). Indeed, appressoria are produced in leaves instead of hyphopodia.

The differentiation of melanized appressoria is a key step in the process of cereal leaf infection by *Magnaporthe oryzae* (Howard and Valent, 1996). Mutants unable to synthesize 1,8‐dihydroxynaphthalene (DHN)‐melanin (e.g., *buf1* mutants) fail to generate the appressorial turgor pressure required for successful penetration of the leaf surface (Chumley and Valent, 1990). However, such mutants are not compromised in their ability to form hyphopodia and can penetrate intact roots (Sesma and Osbourn, 2004). Tucker *et al*. (2010) also identified *M. oryzae* mutants with organ‐specific phenotypes, including five mutants unable to infect leaves and one mutant unable to infect roots. Their results suggest distinct genetic requirements for appressorium, hyphopodium, and preinvasive hyphae differentiation. Similarly, in *F. oxysporum, fow1* mutants are unaffected in their ability to penetrate root epidermal cells but cannot spread further toward the vascular tissues (Inoue *et al*., 2002). *M. oryzae* mutants of the homologous gene also show defects in root colonization but are completely unaffected during leaf infection (Sesma and Osbourn, 2004).

### 3.4 Effectors and suppression of organ defences

Considering a whole‐plant pathogen, different repertoires of effectors are potentially secreted in plant organs as plant defences and nutrients segregate spatially (see above). Tassels and leaves are two distinct organs of maize that can be infected by *U. maydis*. Skibbe *et al*. (2010) found that *U. maydis* infection of seedling leaves, mature leaves, and tassels causes organ‐specific transcriptional changes in both the pathogen and the host. Of particular interest, *U. maydis* genes encoding secreted proteins are differentially expressed depending on the colonized maize organ. Therefore, Skibbe *et al*. hypothesized that the fungus secretes effectors that act in an organ‐specific manner. Later, Schilling *et al*. (2014) presented the identification and functional characterization of 20 putative organ‐specific *U. maydis* effector genes. *U. maydis* deletion strains were generated for these genes and tested for infectivity of maize seedling leaves and tassels. This approach identified 11 effector genes required for the full virulence of *U. maydis* (e.g., um02239 and um05439; Table 1); nine of them were organ specific, as virulence was only affected in one of the tested plant organs. These results demonstrate that individual fungal effector proteins can contribute to fungal virulence in an organ‐specific manner.

The presence or absence of effector targets could be a partial explanation for the organ specificity of effectors. As a more obvious example, many effectors have been shown to localize to chloroplasts and alter various processes (Serrano *et al*., 2016; Lorrain *et al*., 2018; Pecrix *et al*., 2019). Because these organelles are not present in nonphotosynthetic organs, pathogenic effectors targeting chloroplast‐specific processes (e.g., photosynthesis) would appear useless when infecting such organs and thus might not be expressed, leading to organ specificity of the effector repertoire.

### 3.5 Genomics and evolution

Organ and tissue specificity of pathogen virulence is putatively driven by genome evolution. Comparative analyses of genomes between pathogenic strains infecting various organs or tissues may unravel organ‐ and tissue‐specific marker genes. This has been investigated in *Xanthomonas* by distinct research groups, where tissue‐specific genetic determinants such as *hpaA*, *xpsD* (Lu *et al*., 2008), and *ecpA* (Zou *et al*., 2012) have been identified by comparing the genomes of *Xanthomonas oryzae* pv*. oryzicola* and *X. oryzae* pv*. oryzae*, which are nonvascular and vascular pathogens of rice, respectively. In filamentous pathogens, genomic determinants of wood colonization have been identified through the genomic comparison of closely related pathogens. Indeed, by comparing the genomes of *Mycosphaerella populorum* (a stem pathogen colonizing wood and causing cankers) and *M. populicola* (a minor leaf pathogen causing leaf spots), Dhillon *et al*. (2015) highlighted that differences in gene content and expression, including a unique horizontally transferred secondary metabolite gene cluster, were responsible for wood adaptation. Similarly, the genomes of *Valsa mali* and *Valsa pyri* have been contrasted with those of related cereal pathogens, and a secondary metabolites gene cluster was suggested to be potentially responsible for the ability of these two pathogens to colonize woody bark (Yin *et al*., 2015). Thanks to the increase of genome availability, further discoveries about determinant factors of structural specificity may be unravelled.

Accessory chromosomes of filamentous plant pathogens, also called conditionally dispensable chromosomes, can be lost without affecting the vegetative development of the organism, compared to core chromosomes (van Dam *et al*., 2017). However, studies conducted on accessory chromosomes show that they sometimes represent a crucial component of microbial virulence, being an absolute requirement for plant infection (Ma *et al*., 2013; Vlaardingerbroek *et al*., 2016). Such chromosomes have been found in various species, such as *Alternaria* spp., *F. oxysporum*, *L. maculans*, *Mycosphaerella graminicola*, and *N. haematococca* (Bertazzoni *et al*., 2018). In multiple *F. oxysporum* formae speciales, accessory chromosomes include genes coding for secreted‐in‐xylem (SIX) effectors (van Dam *et al*., 2017). When one or more accessory chromosomes are acquired by another *F. oxysporum* forma specialis through horizontal chromosome transfer, it renders it virulent on the original host, that is, the host on which the accessory chromosomes were required for infection. Because of the importance of accessory chromosomes in dictating host specificity, it is tempting to speculate that they are also involved in structural specificity. Indeed, while the biotrophic stage of *F. oxysporum* includes the penetration of the fungus through the epidermis into the root and colonization of the cortex and endodermis, its subsequent necrotrophic stage is observed during colonization of the vascular bundles (Ma, 2014). As suggested by van Dam *et al*. (2017), the genetic determinants behind the differences in disease symptoms caused by distinct *Fusarium* isolates (root rot vs. wilt) may well be associated with the repertoire of effectors harboured by those accessory chromosomes.

## 4 CONCLUSION

**FIGURE 3:**
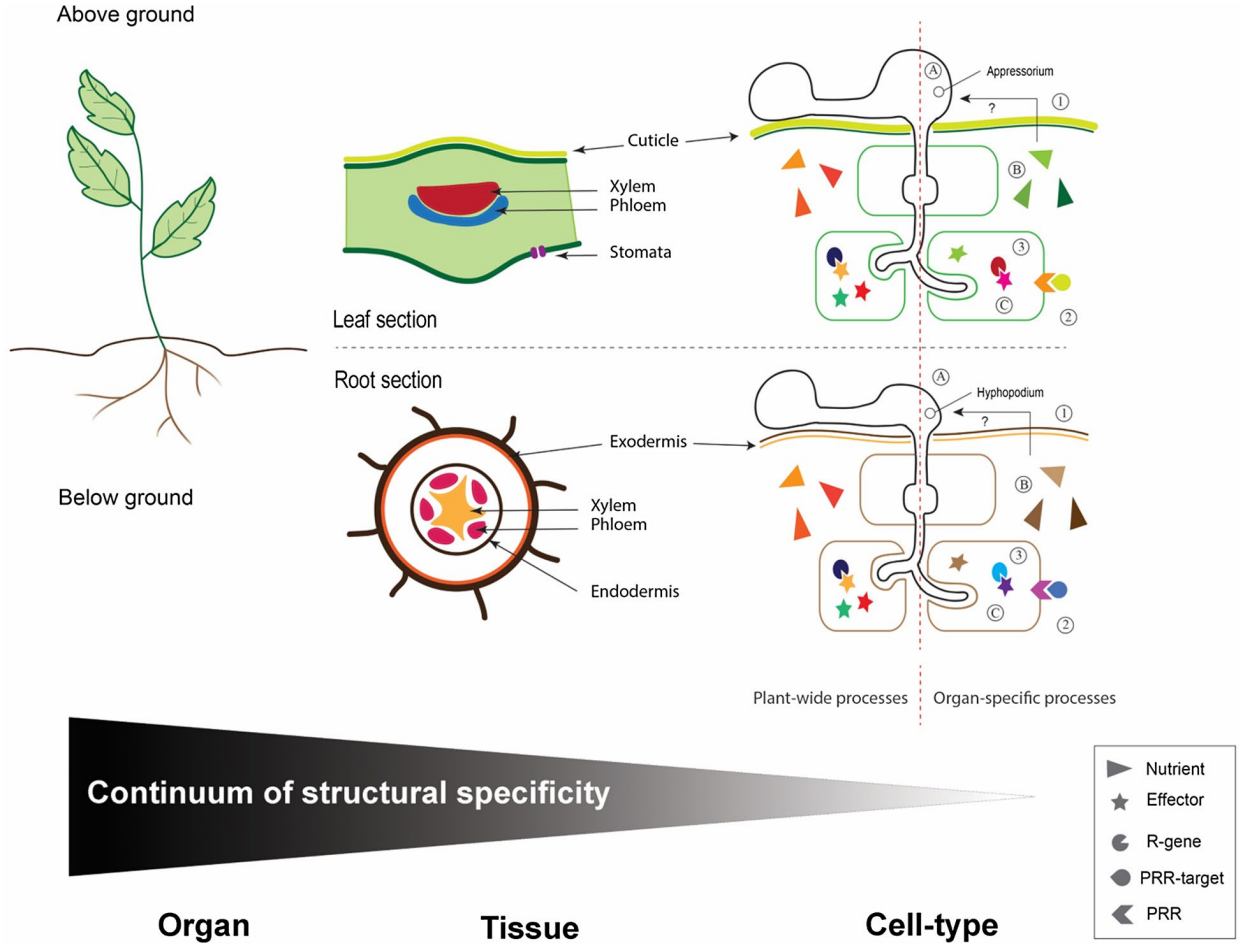
Various levels of structural specificity in a plant–filamentous pathogen interaction. The first level of structural specificity is at the organ level. Organs are often divided as being above or below ground, and encompass well‐known structures (leaves, stems, flowers, and fruits vs. roots and tubers). Specificity can be also restricted to tissues (e.g., vascular tissues, dermal tissues) or cell types (e.g., sieve elements, guard cells). Various components of each of the plant defence layers appear to segregate and act differently: (1) preformed defences (e.g., cuticle in leaf vs. exo/endodermis in roots), (2) PRR‐mediated immunity (PMI), and (3) NLR‐mediated immunity (NMI; both PMI and NMI showing specialist and generalist receptors). Filamentous pathogen processes also exhibit structural specificity: (A) morphological and developmental adaptation, (B) nutrient availability/acquisition, and (C) effector waves. The black arrow depicts the putative influence of organ‐specific nutrients on the morphological and developmental adaptation of pathogens

Almost 50 years ago, Graniti noted that “biochemical and physiological research on [organ‐specificity] is needed for most plant pathogens showing electivity for host tissues and organs, a field which appears to have been largely neglected by plant pathologists” (Graniti, 1976). Nowadays, studies on structural specificity in plant–microbe interactions are attracting more interest, and a significant body of knowledge has been acquired over the past few years. It appears clear that plant resistance as well as pathogen virulence contribute to degrees of structural specificity, whether in terms of either tissue or organ specificity (Figure 3). This specificity is an important determinant in the outcome of a plant–pathogen interaction where both partners appear to be involved. However, it can be difficult to distinguish between host versus pathogen contribution to organ specificity. Does one of the two partners dominate the outcome, whether the host tissue/organ or the microbe, or is it truly a combination of the two?

In plants, preformed barriers and processes, PMI, and NMI all spatially segregate among plant organs and tissues. Compared to shoots, roots are constantly exposed to soilborne microbes and it would be wasteful to constitutively express defence‐related components in this organ. Indeed, as resistance processes have a non‐negligible energy cost, plants may differentially allocate this energy depending on the necessity. Moreover, the defence status is dependent on its nutritional status. In a nutrient‐rich environment, roots might have a stronger ability to defend themselves, while in a poor environment, roots might be less able to defend themselves. In pathogens, morphology and effector repertoires, driven by nutrient availability and/or developmental stages, are also distinct when it comes to the affected organ or tissue. To go further in the potential organ specificity of pathogen morphology, a recent publication pointed out the compartmentalization of *S. sclerotiorum* infectious hyphae. Those different compartments cooperate through resource allocation and division of labour to facilitate a host colonization (Peyraud *et al*., 2019). Thus, these findings suggest that infectious hyphae themselves are constituted of different tissues and could thus be considered as organs. It is worth thinking that these organs contribute differentially to structural specificity.

In this review, we focused on differences between organ and tissue, although there are many commonalities. Furthermore, it is important to note that the finer level of structural specificity, cell type specificity, is only starting to be investigated in both plants and pathogens (Matei *et al*., 2018). Technical limitations are being resolved as approaches such as laser‐capture microdissection, flow cytometry, and single‐cell transcriptomics are being improved.

Currently, the knowledge landscape is somewhat fragmented. The demonstration that a particular genotypic combination of host and pathogen is incompatible in a given organ (e.g., in a foliar assay) should not lead to the conclusion that a plant genotype is resistant to that particular isolate in all of its tissues. However, additional studies are needed to improve our understanding of this phenomenon. Such research could rely on the identification of cell‐type‐specific effectors and the investigation of structural specificity of both PMI and NMI. Towards this idea, accessible pathosystems exhibiting structural preferences are needed to address these open questions straightforwardly and allow the identification of general patterns and concepts. Examples of such systems include *Magnaporthe* species capable of infecting both roots and shoots (Sesma and Osbourn, 2004; Marcel *et al*., 2010), as well as various *Fusarium* or *Phytophthora* species (*P. capsici*, *P. palmivora*, and *P. infestans*). Systematic approaches to decipher the organ‐specific expression patterns of both plant defence components and microbial effectors should lead the way.

## Data Availability

Data sharing is not applicable to this article as no new data were created or analysed in this study.
